# AI-based diagnostic tools for oral cancer: A systematic review

**DOI:** 10.6026/973206300213324

**Published:** 2025-09-30

**Authors:** Sindhoori Goud, Anuj Singh Parihar, Anuj Mishra, Tanu Sahney, Sujit Anil Vyavahare, Madhura Govind Titar, Julee Mandal

**Affiliations:** 1 Department of Conservative Dentistry and Endodontics, Mallareddy Institute of Dental Science, Mallareddy Viswavidyapet, Hyderabad, Telangana, India; 2 Department of Periodontology, People's Dental Academy, Bhopal, Madhya Pradesh, India; 3 Department of Oral Medicine and Radiology, Sardar Patel Post Graduate Institute of Dental and Medical Sciences, Lucknow, Uttar Pradesh, India; 4 Department of Periodontology, Sardar Patel Post Graduate Institute of Dental and Medical Sciences, Lucknow, Uttar Pradesh, India; 5 Department of Oral and Maxillofacial Surgery, MGM Dental College and Hospital, Kamothe, Navi Mumbai, Maharashtra, India; 6 Department of Prosthodontics and Crown and Bridge, MGM Dental College and Hospital, Kamothe, Navi Mumbai, Maharashtra, India; 7 Department of Dentistry, Kalinga Institute of Dental Sciences, Kalinga Institute of Industrial Technology (KIIT) Deemed to be University, Bhubaneswar, Odisha, India

**Keywords:** Artificial intelligence, oral cancer, deep learning, diagnostic accuracy, convolutional neural networks, image analysis

## Abstract

Artificial intelligence (AI) as a means of enhancing and improve the early detection and diagnosis of oral cancer. While traditional diagnostic methods are effective, they are inherently subjective, not widely accessible, and often result in detection only at later stages of disease progression. The literature over the past few years has importantly identified that AI models, in particular deep learning and convolutional neural networks, displayed high diagnostic accuracy on clinical, histopathological and optical imaging data. However, challenges exist in the form of variability in the data sets, limited external validation in clinical practice and or interpretability of AI models for clinical use. In conclusion, AI presents a compelling opportunity as a supportive adjunct for oral oncology, although standardized validation and real-world implementation should occur before widespread utilization.

## Background:

Oral cancer is a significant public health issue on a global scale and has a particularly high incidence in South Asian and Southeast Asian countries due to the high level of tobacco, alcohol, and betel quid use in these regions [1]. The GLOBOCAN 2020 database reported that lip and oral cavity accounted for 377,713 new cases and 177,757 deaths worldwide, signifying a substantial disease burden for society [2]. The prognosis for oral cancer depends heavily upon the stage at diagnosis, with survival exceeding 80% in early-stage cancers that fall below 40% in late-stage cancers [3]. Conventional diagnostic approaches, including clinical visual inspection, toluidine blue staining, brush biopsy, scalpel biopsy, and confirmatory pathology often effectively diagnose oral cancer. However, many of these diagnostic approaches are diagnostic, subjective, inter-observer variability, and dependent on highly skilled pathologists [4]. Subtle malignant lesions, including leukoplakia, and erythroplakia, may lack significant clinical features and difficult to diagnose early, so late presentation is often common [5]. In low resource settings, all the above conditions will be exacerbated by insufficient scales and a shortage of qualified professionals [6]. Artificial intelligence (AI) has become an exciting mechanism to increase diagnostic accuracy in oncology through the processing of very large amounts of information contained within medical images and clinical data [7]. Machine learning (ML) and its specialized field, deep learning (DL), have specifically been demonstrated, with expertise in convolutional neural networks (CNNs), to characterize malignant features, classify lesions, and estimate prognosis based on histopathological slides and clinical photographs [8]. A number of reports suggest that artificial intelligence (AI) models may have comparable differentiate oral squamous cell carcinoma from benign lesions compared to clinical experts [9]. The well-established scalability, objectivity, and prospective utility in limited-resource environments of AI-based diagnostic systems offer promising potential for implementing population screening programs [10]. Certainly, many of these reports are limited by their dataset being solely from their own institution, and the existing barriers around external validation, standardization, and clinical adaptation must also be acknowledged as limitations when reviewing the encouraging literature surrounding the AI diagnostic at scale [11]. Therefore, it is of interest to narratively review and synthesize recent evidence on the application of AI in oral cancer diagnosis.

## Evidence from recent literature:

In recent years, there have been a growing number of studies investigating artificial intelligence (AI) in the area of oral oncology in clinical imaging, histopathology, cytology, and optical technologies. Across studies, convolutional neural networks (CNNs), support vector machines (SVMs), and ensemble algorithms have consistently shown good diagnostic efficacy in detecting oral cancer and precancerous lesions. Within clinical imaging, AI systems are extremely promising for the identification of oral malignancies from intraoral photographs and optical technologies. Kouketsu *et al.* (2024) applied a Single Shot Multibox Detector (SSD) model to intraoral photos with sensitivity as high as 93.9% and specificity of 81.2% [14]. Jerjes *et al.* (2024) used AI augmented optical coherence tomography (OCT) for identification of lesions and reported sensitivity between 75-100% and specificity between 71-100% depending on type of lesion [16]. Most notably, García-Pola *et al.* (2021) examined various algorithms applied to cytology and imaging datasets and ultimately highlighted the potential of AI technology into clinical workflows [20]. AI applications informed by histopathology have been notably successful due to the ability to obtain cellular and tissue-level data. For example, Khanagar *et al.* (2021) used CNN-based models for image classification of histopathologic images achieving diagnostic accuracy rates of 89-100% and reports of a high sensitivity of 99.26% and specificity of 96.43% [15]. Alabi *et al.* (2021) demonstrated that machine learning algorithms have sound fidelity when processing histopathological data [7]. In addition, Li *et al.* (2024) achieved balanced performance across multiple AI models and varied diagnostic classes, showing measure of sensitivity of 89.9% and specificity of 89.2% [19]. The body of work included hybrid and ensemble approaches in machine learning. In particular, Tobias *et al.* (2022) discussed the extractive properties of CNN as applied to clinical photographs, providing important evidence to facilitate the potential future integration of clinical photographs into diagnostic pipelines [2]. Likewise, Wuttisarnwattana *et al.* (2024) and Zayed *et al.* (2024) studied the application of AI models across clinically and histopathologically based datasets that were unique and heterogeneous by combining multimodal information from clinical and histopathological domains [11, 12]. While the literature continues to evolve in this direction due to numerous publications, clear limitations also exist. For instance, many studies published are based off small dataset sizes obtained from a single institution, and not generalizable, limiting external validity [13, 14, 16 and 19]. The literature highlights a lack of standardization in imaging protocols and annotation which leads to inconsistencies in outcome reporting [21]. Additionally, very few studies used explainability tools like saliency maps or Grad-CAM. This is crucial for clinician trust-based uptake of AI-assisted diagnostic algorithms in clinical practice. In particular literature gaps in interpretability do not explore explaining models that include saliency maps or Grad-CAM [23]. Overall, the evidence shows that AI can show promising levels of diagnostic accuracy in orally cancer detection across modalities and types of data, particularly deep learning models using convolutional neural networks (CNN), representation or quantitative data extracted from various datasets may need to be explored along this line. While this is promising and encouraging together with acknowledging the even use of AI in even small reviews, good intentions of studies of AI, and potential is encouraging, a lot of the studies are in their proof of concept, and promise but results need to be tested in larger multicenter trials, real-world patients, and to ensure they can be explained readily to patients to be routinely embedded in their clinical workflow, how we use AI and its uptake more effectively if we understand limitations and communicate them readily to patients to improve utilization outcomes. Of the ten included studies in Table 1 (see PDF), only four (Li *et al.* [19], Jerjes *et al.* [16], García-Pola *et al.* [20], and Kouketsu *et al.* [14]) performed external validation, while the remaining studies relied on internal validation approaches such as k-fold cross-validation or holdout testing.

## Discussion:

This systematic review presents an overview of recent findings regarding the application of artificial intelligence (AI) in the diagnosis of oral cancer exclusively including verified peer-reviewed articles published on PubMed and Scopus [10]. The literature supports that AI diagnostic performance, predominantly using deep learning models such as convolutional neural networks (CNNs), is consistently high for a variety of imaging modalities including clinical photographs, histopathology images and optical coherence tomography (OCT) [11]. These investigations in the literature generally advocate for the increasing role of AI as a remarkable adjunct to ordinary clinical examination and pathology, particularly with respect to diagnostic performance and decreasing bias [12]. Various articles evaluated in this review present AI models with diagnostic performance over 90%. For instance, Kouketsu *et al.* (2024) utilized a single-shot multibox detector (SSD) to identify oral malignancies from intraoral photographs resulting in a sensitivity and specificity of 93.9% and 81.2%, respectively [14]. Additionally, Khanagar *et al.* (2021) discussed the implementation of CNN histopathological classification models with sensitivity of 99.26% and precision was greater than 89%, illustrating the capability of AI to interpret very detailed diagnostic information [15]. Furthermore, Jerjes *et al.* (2024) presented a case of improving diagnostic accuracy with optical coherence tomography (OCT), further extending the application of AI into the area of our understanding of non-invasive diagnostic performance, reporting a sensitivity between 75% and 100% depending on lesion type [16]. These results highlight the potential of AI to surpass or, at the very least, perform comparably to human specialists for specific diagnostic functions, particularly if trained on abundant high-quality annotated data. The proposition of AI is not merely the capacity to diagnose, but the potential broad reach of its application, the ability to maintain a standardized objective evaluation, and its applicability in a resource-poor context 17]. In situations where an expert oral pathologist or adequate histopathology infrastructure is unavailable, AI can be employed as a first assessment tool to assess high-risk lesions in order to later proceed to a detailed histopathological evaluation [18]. Evidence provided by Li *et al.* (2024) and Garcia-Pola *et al.* (2021) have highlighted this ability with case studies demonstrating how AI models can be integrated into clinical pathways for early detection, which in turn could potentially help reduce diagnostic delays and improve patient outcomes [19, 20]. The finding from the present review has wide support from studies conducted by Veeraraghavan *et al.* and Kavyashree *et al.* both of which emphasized the great diagnostic capacity in CNN-based models [8, 9]. In contrast to these prior reviews, the current review included studies from several 2024 publications and provides in-depth analysis of bizarre validation strategies, demonstrating a continued over-reliance on internal testing without sufficient external validation in the majority of AI models.

Still, even with these encouraging advances, there are multiple crucial limitations and gaps this review finds that need to be bridged before AI can be seamlessly integrated into routine clinical practice. To begin with, the diversity of datasets employed within the included studies varied in size, resolution, imaging modality and annotation protocols undermines generalizability of model performance [21]. Lack of publicly available, standardized repositories of images compiles benchmarking as well as external validation. Fewer than half of the studies conducted external validation in independent datasets, which is essential for proving model robustness across different populations and clinical environments [22]. Second, the issue of model interpretability remains a substantial barrier to clinical adoption. Most AI models, especially deep learning architectures, function as "black boxes," with limited transparency in decision-making logic. Although some studies have begun incorporating visualization techniques such as Grad-CAM and saliency maps, these tools remain largely underutilized and inconsistently reported. For AI to be trusted by clinicians, especially in high-stakes decisions like cancer diagnosis, interpretability and explainability must be core design features [23]. Third, the lack of real-world deployment studies is a significant shortcoming. None of the included studies conducted randomized clinical trials or head-to-head comparisons between AI-assisted and traditional diagnostic pathways. Moreover, ethical concerns such as algorithmic bias, data privacy and informed consent were not addressed in detail in most reports. These factors will need careful attention as AI transitions from research settings to clinical application [24]. Evidence affirms that AI holds substantial potential in oral oncology diagnostics, especially in enhancing early detection, reducing human error and supporting clinical decision-making. Rather than replacing clinicians, AI should be viewed as a complementary tool that augments human expertise, facilitates rapid screening and democratizes access to high-quality diagnostics.

## Conclusion:

Artificial intelligence, especially deep learning models like convolutional neural networks, has demonstrated strong diagnostic accuracy in the detection of oral cancer across various imaging modalities. Despite promising results, challenges such as dataset variability, lack of external validation and limited clinical integration remain significant barriers to real-world application. Future efforts must prioritize standardized multicenter validation and clinically interpretable AI systems to support safe, scalable implementation in routine oral oncology diagnostics.

## References

[1] Ahmed N (2021). Biomed Res Int..

[2] Tobias MAS (2022). Oral Oncol..

[3] Mahmood H (2021). Br J Cancer..

[4] Hung M (2020). World J Clin Oncol..

[5] Kawamura K (2021). Gan To Kagaku Ryoho..

[6] Krishna AB (2020). J Oral Maxillofac Pathol..

[7] Alabi RO (2021). Artif Intell Med..

[8] Veeraraghavan VP (2024). J Craniofac Surg..

[9] Kavyashree C (2024). Healthc Anal..

[10] Ilhan B (2020). J Dent Res..

[11] Zayed SO (2024). BMC Oral Health..

[12] Wuttisarnwattana P (2024). Stud Health Technol Inform..

[13] Mahmood H (2020). Oral Oncol..

[14] Kouketsu A (2024). Head Neck..

[15] Khanagar SB (2021). Diagnostics..

[16] Jerjes W (2024). J Clin Med..

[17] Kar A (2020). J Oral Pathol Med..

[18] Alhazmi A (2021). J Oral Pathol Med..

[19] Li JW (2024). Head Neck..

[20] García-Pola M (2021). Cancers (Basel)..

[21] Seneviratne CJ (2020). Crit Rev Microbiol..

[22] Yoshida K (2019). Photobiomodul Photomed Laser Surg..

[23] Ennab M (2024). Front Robot AI..

[24] El Arab RA (2025). Healthcare (Basel)..

